# Parallel-Batch Scheduling and Transportation Coordination with Waiting Time Constraint

**DOI:** 10.1155/2014/356364

**Published:** 2014-04-15

**Authors:** Hua Gong, Daheng Chen, Ke Xu

**Affiliations:** College of Science, Shenyang Ligong University, Shenyang 100159, China

## Abstract

This paper addresses a parallel-batch scheduling problem that incorporates transportation of raw materials or semifinished products before processing with waiting time constraint. The orders located at the different suppliers are transported by some vehicles to a manufacturing facility for further processing. One vehicle can load only one order in one shipment. Each order arriving at the facility must be processed in the limited waiting time. The orders are processed in batches on a parallel-batch machine, where a batch contains several orders and the processing time of the batch is the largest processing time of the orders in it. The goal is to find a schedule to minimize the sum of the total flow time and the production cost. We prove that the general problem is NP-hard in the strong sense. We also demonstrate that the problem with equal processing times on the machine is NP-hard. Furthermore, a dynamic programming algorithm in pseudopolynomial time is provided to prove its ordinarily NP-hardness. An optimal algorithm in polynomial time is presented to solve a special case with equal processing times and equal transportation times for each order.

## 1. Introduction 

A supply chain is made of all the stages of value creation such as supply, production, and distribution. In logistics management, there are usually transportation of raw materials or semifinished products and distribution of products. Researches on supply chain management focus on developing strategies to help companies to improve optimal chain-wide performance by proper coordination of the different stages of supply chains. For an order, transportation of raw materials or semifinished products and production are two key operations in the supply chain system. A scheduling problem with the limited waiting time occurs when the processing of orders that finished transportation has to start in a given time. There are several industries where the transportation and production scheduling problem is influenced by the limited waiting time. Examples include fresh food and chemical and automobile industries. For instance, in case of fresh food production, canning operation must follow cooking operation to ensure freshness after transportation of raw materials. Additional applications can be found in the advanced automobile manufacturing environments such as just-in-time systems. Auto parts of orders are transported from suppliers to producers for further assembling. The producers must process the orders arrived at the factory in a given time in order to decrease the inventory level and increase the production level. This situation is relevant to logistics and supply chain management and specifically addresses how to balance the transportation rate of raw materials and the production rate.

Motivated by the aforementioned problem in logistics management, this paper describes a coordinated model for scheduling transportation of raw materials or semifinished products and parallel-batch production with waiting time consideration. There is a set of orders located at the different suppliers. The orders are transported by some vehicles to a manufacturing facility to be further processed. But only one order can be transported at a time. Each order arriving at the facility must be processed in its given waiting time. The orders are processed in batches on a parallel-batch machine, where a batch contains several orders and the processing time of the batch is the largest processing time of the orders in it. The goal is to find a schedule to minimize the sum of the total flow time and the production cost.

Production scheduling problems with transportation considerations have been studied by a number of researchers. The parallel-batch machine scheduling problems have been previously studied in other manufacturing systems, especially burn-in operations in the very large-scale integrated circuit manufacturing (e.g., [1–3]). We briefly discuss some work related to integrated production scheduling and transportation decisions. Some research (see [4–7]) has been done for two machine flow shop problems featuring transportation of semifinished products and batching processing. We mention the studies in [4–7]; however, the problems there do not consider parallel-batch production or they consider another kind of batching with a constant processing time. The models in [8, 9] consider the coordinated scheduling problems with two-stage transportation and machine production that incorporate the scheduling of jobs and the pickup of the raw materials from the warehouse and the delivery arrangement of the finished jobs. The above scheduling problems do not consider the waiting time constraints. Another line of production scheduling models with transportation decisions focuses on the delivery of finished jobs to customers with a limited number of vehicles. Interested readers for the delivery coordination are referred to the recent review in [10]. The parallel-batch machine scheduling problems with batch delivery are considered in [11, 12]. All of the papers reviewed in this section deal in some way with the coordination of production scheduling and transportation at logistics and operations area, but none of them addresses and deals with the problem from the limited waiting time point of view. We not only consider the scheduling problem involving transportation capacity and transportation times but also take into account the order waiting time constraint and the batch capacity. Here, the coordination of batch processing, transportation, and waiting time constraints is a decision about whether or not to reduce production cost and improve machine utilization.

The remainder of this paper is organized as follows. In the next section, we introduce the notation to be used and describe the model. In Section 3, we analyze the optimal properties of the problem and prove strong NP-hardness of the general problem. In Section 4, a dynamic programming algorithm is provided to solve the problem with equal processing times and we prove that this case is NP-hard in the ordinary sense.  Section 5 deals with a case with equal processing times and equal transportation times and shows how to find the optimal schedule. The last section contains a conclusion and some suggestions for future research.

## 2. Description and Notation

Our problem is formally stated as follows. Given a set of *n* orders (including some semifinished jobs or raw material in one order), *J* = {*J*_1_,…, *J*_*n*_}, which are located at the different suppliers. The orders need to be transported to a manufacturing facility and processed on a single parallel-batch machine. Only *m* vehicles are available for transporting the orders, which can load only one order in one shipment. The vehicles are initially located at a transportation center. Let *t*_*j*_ denote the transportation time of order *J*_*j*_ from the supplier to the manufacturing facility. The transportation time from the factory back to the center is negligible. In the production part, each order *J*_*j*_ requires a processing time of *p*_*i*_ on the parallel-batch machine. The preemption of orders is not allowed. The parallel-batch machine can process a number of orders simultaneously as a batch as long as the total number of orders in the batch does not exceed the machine capacity *c*. The processing time of a batch is the longest processing time of all orders in it. Orders processed in the same batch have the same completion time, that is, their common start time (the start time of the batch in which they are contained) plus the processing time of the batch. Suppose that loading and unloading times are included in the transportation times and the processing times of orders. Associated with each order is *w*_*j*_, which is the waiting time of an order for a period time from arriving at the machine to starting its processing on the machine. Here, *w*_*j*_ is restricted by a constant *W* where max⁡_*j*_ *p*_*j*_ ≤ *W* ≤ ∑*p*_*j*_, such that the schedule has a feasible solution at least. Each batch requires a processing cost on the parallel-batch machine which reflects the production cost. The goal is to find a feasible schedule to minimize the sum of the total flow time and the total processing cost.

*Decision Variables*

Consider the following:

 
*T*_*u*_: the total running time of vehicle* u*,
 
*r*_*j*_: the arrival time of order *J*_*j*_ on the machine,
 
*s*_*j*_: the starting time of order *J*_*j*_ on the machine,
 
*w*_*j*_: the waiting time of order *J*_*j*_ on the machine, *w*_*j*_ = *s*_*j*_ − *r*_*j*_ ≤ *W*,
 
*B*_*l*_: batch* l*,
 
*b*_*l*_: the number of orders in *B*_*l*_,
 
*P*(*B*_*l*_): the processing time of *B*_*l*_ on the machine, *P*(*B*_*l*_) = max⁡_*j*∈*B*_*l*__{*p*_*j*_},
 
*S*_*l*_: the starting time of *B*_*l*_ on the machine,
 
*D*_*l*_: the completion time of *B*_*l*_ on the machine,
 
*C*_*j*_: the flow time of order *J*_*j*_ on the machine,
 
*x*: the number of batches,
 
*X*(*x*): the total processing cost, a nondecreasing function of* x*,
 
*F*(*π*) = ∑_*j*=1_^*n*^ *C*_*j*_ + *X*(*x*): the objective function of a schedule *π*.

We follow the commonly used three-field notation, *α*|*β*|*γ*, introduced to denote the problem under study. The problem is denoted by *Vm* → batch|*w*_*j*_|∑*C*_*j*_ + *X*(*x*)  where  *α*  field indicates that the orders are first transported by *m* vehicles and then processed on the parallel-batch machine.  *β*  field describes the order restrictive requirement, and  *γ*  defines the objective function to be minimized.

Specifically, the tradeoff between processing and transportation gives rise to the sequence of transportation and the batch composition decisions. The schedule may produce more batches in order to satisfy the waiting time requirements of orders. This leads to the increase of the total processing cost on the machine. On the other hand, if we create very few batches, then we may not obtain a feasible schedule due to waiting time constraint. Therefore, minimizing of the objective for the problem is achieved a feasible schedule for sequencing and batching to tradeoff the total flow time and the processing cost. The following example gives an illustration of this observation.

**Example 1**

Consider a set of 8 orders with their transportation times and processing times as shown below for the problem  *Vm* → batch|*w*_*j*_|∑*C*_*j*_ + *X*(*x*).

Suppose *m* = 2,  *c* = 4,  *W* = 4, and *X*(*x*) = 3*x* (see Table 1).

One vehicle transports the orders in turn: *J*_1_,  *J*_3_,  *J*_5_, and *J*_7_. Another vehicle transports the orders in turn: *J*_2_,  *J*_4_,  *J*_6_, and *J*_8_. The constructed schedule *π* consists of three batches, *B*_1_ = {*J*_1_, *J*_2_, *J*_3_, *J*_4_},  *B*_2_ = {*J*_5_, *J*_6_}, and *B*_3_ = {*J*_7_, *J*_8_}, and has an objective value of 85. If another constructed schedule *π*′ contains two batches *B*_1_ = {*J*_1_, *J*_2_, *J*_3_, *J*_4_},  *B*_2_ = {*J*_5_, *J*_6_, *J*_7_, *J*_8_}, then the waiting time of the order *J*_5_ is 5. Although schedule *π*′ has a smaller processing cost, it is not feasible. From these two schedules, we see that a minimized objective can be obtained from a proper combination of the sequence of transportation and the batching of processing under the waiting time constraints.

## 3. The Problem *Vm* → batch|*w*_*j*_|∑*C*_*j*_ + *X*(*x*)

In this section, we analyze two properties of the scheduling problem and prove that* Vm*→batch|*w*_*j*_|∑*C*_*j*_ + *X*(*x*) is NP-hard in the strong sense by a reduction from the 3-Partition Problem, which is well known to be NP-hard in the strong sense [13].

*3-Partition Problem (3-PP)*. Given 3*h* items, *H* = {1,2,…, 3*h*}, each item *i* ∈ *H* has a positive integer size *a*_*i*_ satisfying *a*/4 < *a*_*i*_ < *a*/2 and ∑_*i*=1_^3*h*^ *a*_*i*_ = *ha*, for some integer *a*. The question asked is whether there are *h* disjoint subsets *H*_1_, *H*_2_,…, *H*_*h*_ of *H* such that each subset contains exactly three items and its total size ∑_*i*∈*H*_*j*__ *a*_*i*_ = *a*, *j* = 1,2,…, *h*.

**Lemma 2**

For the problem* Vm*→batch|*w*_*j*_|∑*C*_*j*_ + *X*(*x*), there exists an optimal schedule without idle time between orders on any vehicle.

The proof of Lemma 2 is straightforward and is omitted.

The following result describes a candidate set of possible processing times.

**Lemma 3**

For the problem* Vm*→batch|*w*_*j*_|∑*C*_*j*_ + *X*(*x*), there exists an optimal schedule in which each processing on the parallel-batch machine is made either at the arrival time of an order on the machine or immediately when the machine becomes available.

**Proof**

Assume that there is a process which is scheduled neither at the arrival time of an order nor at a time when the machine becomes available. This process can be changed to the latest earlier time which fits either of those conditions. Since the same orders can be processed at that earlier time and there are no additional processes, the objective value is not increased.

**Theorem 4**

The problem* Vm*→batch|*w*_*j*_|∑*C*_*j*_ + *X*(*x*) is strongly NP-hard even if *m* = 2.

**Proof**

To show the NP-hardness of the scheduling problem, we establish the following polynomial time reduction from the 3-PP. Given a 3-PP instance, we construct an instance for *Vm* → batch|*w*_*j*_|∑*C*_*j*_ + *X*(*x*) as follows.

*Number of Orders*. Consider

(1) $$\begin{array}{c} n=7\;h+7, \end{array}$$

*Ordinary Orders*. Consider

(2) tj=⌈j6⌉a⌊(1/2)(j+1)⌋,  pj=2(⌈j6⌉+1)a,           j∈H={1,2,…,6h}.

*Auxiliary Orders*. Consider

(3) tj=2(j−6h)a, j∈G={6h+1,…,7h}pj=p6h+i=2(i+1)(a3i−2+a3i−1+a3i),       j∈G={6h+1,…,7h},tj=0, pj=2a, j∈Y={7h+1,…,7h+7},

*Machine Capacity*. Consider

(4) $$\begin{array}{c} c=7, \end{array}$$

*Limited Waiting Time*. Consider

(5) $$\begin{array}{c} W=2ah, \end{array}$$

* Processing Cost Function*. Consider

(6) $$\begin{array}{c} X\left(x\right)={\left[\frac{5}{3}a\left(h+1\right)\left(h+2\right)\left(h+3\right)\right]}^{x-h}, \end{array}$$

*Threshold Value*. Consider

(7) $$\begin{array}{c} y=4a\left(h+1\right)\left(h+2\right)\left(h+3\right). \end{array}$$

Clearly, in a solution to this instance of *Vm* → batch|*w*_*j*_|∑*C*_*j*_ + *X*(*x*) with the objective value not exceeding* y*, we first prove the following properties: (1) since *t*_*j*_ = 0, for *j* ∈ *Y*, all the orders of *Y* as the first batch must be processed on the machine in the time interval [0, 2*a*]; (2) there will be exactly *h* + 1 batches in the optimal schedule, and each batch contains 7 orders. Suppose that there are* q* (more than *h* + 1) batches in the schedule. Then 7*h* + 7 orders need at least *h* + 1 batches due to *c* = 7. If *q* = *h* + 2, then the total processing cost is *X*(*x*) = *X*(*h* + 2) = [(5/3)*a*(*h*+1)(*h*+2)(*h*+3)]^2^ > *y*. Thus, all the orders of *H* ∪ *G* are divided into *h* batches; that is, all the batches are full.

We will show that the 3-PP instance has a solution if and only if there is a schedule *π* for the scheduling instance such that its objective value of *π* is no more than* y.*

**Figure 1:**
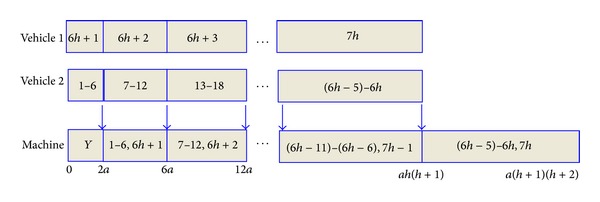
The schedule of Theorem 4.

→Assume that there is a solution to the 3-PP instance, *H*_1_, *H*_2_,…, *H*_*h*_, then there is a schedule to our problem with an objective value of no more than *y*. We construct a schedule *π* for our problem as shown in Figure 1.

In this schedule, two vehicles begin to transport orders at time point 0. All the orders of *Y* are processed first on the machine as the first batch. In the interval [*al*(*l* − 1), *al*(*l* + 1)], vehicle 1 transports the orders of *H*_*l*_,  *H*_*l*_ = {6*l* − 5,  6*l* − 4,…, 6*l*}, vehicle 2 transports *J*_6*h*+*l*_ , for *l* = 1, 2, …, *h*. Since *t*_6*h*+*l*_ = 2*la* and ∑_*j*∈*H*_*l*__ *t*_*j*_ = 2*la*, *J*_6*h*+*l*_  and the last order of *H*_*l*_ arrive at the machine simultaneously, for *l* = 1, 2, …, *h*, then  *J*_6*h*+*l*_ and the corresponding orders of *H*_*l*_ form batch *B*_*l*_. The waiting time of each order in *B*_*l*_ is smaller than *W*. Hence, the starting time of *B*_*l*_ on the machine is *al*(*l* + 1); the completion time of *B*_*l*_ is *a*(*l* + 1)(*l* + 2), for *l* = 0,1, 2, …, *h*. It is easy to see that the above schedule is optimal and the objective value is* y*.

←On the contrary, suppose that there exists a schedule for the constructed instance with the objective value not exceeding* y*.

*Fact  1*. In the schedule, *B*_*l*_ contains an auxiliary order *J*_6*h*+*l*_, for *l* = 1, 2, …, *h*.

Assume that there is some batch *B*_*l*_ that contains more than one auxiliary order. Assume that *B*_*l*_ contains two auxiliary jobs *J*_6*h*+*l*_  and *J*_6*h*+*k*_ ; then there must be some batch *B*_*k*_ only consisting of the ordinary jobs, *k* ≠ *l*. Consider the following two cases.

*Case  1*. If *k* = *l* − 1, then it is shown that *B*_*l*−1_ = {*J*_6*l*−11_,…, *J*_6*l*−6_, *J*_*j*′_ | *j*′ ∈ *H*_*l*_}. The corresponding flow time of each order in *B*_*l*−1_ satisfies *C*_*j*_ = *al*(*l* + 1) + 2*a*, for*j* ∈ *B*_*l*−1_. The flow times of orders in each batch after *B*_*l*−1_ will be greater than *a*(*l* + 1)(*l* + 2) + 2*a*. It is easy to see that ∑*C*_*j*_ = 4*a*(*h* + 1)(*h* + 2)(*h* + 3) + 14(*h* − *l* + 2)*a*, 2 ≤ *l* ≤ *h*. Hence, *F*′ = ∑*C*_*j*_ + *X*(*h* + 1) > *y*.

*Case  2*. If *k* = *l* + 1, then it can be shown that two vehicles must transport two auxiliary orders of batch *B*_*l*_, respectively. Due to the limited waiting time of orders, the completion time of batch *B*_*l*_ on the machine is greater than *a*(*l* + 1)(*l* + 2) + 2*a*. Furthermore, it is easy to see that ∑*C*_*j*_ > 4*a*(*h* + 1)(*h* + 2)(*h* + 3) + 14(*h* − *l* + 1)*a*, 1 ≤ *l* ≤ *h* − 1, which implies *F*′ > *y*.

*Fact  2*. Each batch *B*_*l*_ must contain 6 orders of *H*_*l*_ = {6*l* − 5,6*l* − 4,…, 6*l*} such that ∑_*j*∈*H*_*l*__ *t*_*j*_ = 2*la*, for *l* = 1, 2, …, *h*.

By the above argument, since each batch is full, we can show that one vehicle transports the orders of *H*_*l*_ one by one, and another vehicle transports *J*_6*h*+*l*_  in the time interval [*al*(*l* − 1), *al*(*l* + 1)]. The orders {*J*_6*h*+*l*_ , *H*_*l*_} as *B*_*l*_ are processed on the machine in the time interval [*al*(*l* + 1), *a*(*l* + 1)(*l* + 2)]. It must be true that ∑_*j*∈*H*_*l*__ *t*_*j*_ = 2*la*, for *l* = 1,2, …, *h*. Otherwise, if ∑_*j*∈*H*_*l*__ *t*_*j*_ < 2*la*, then *p*_6*h*+*l*_ = 2(*l* + 1)·(*a*_3*l*−2_ + *a*_3*l*−1_ + *a*_3*l*_) < 2(*l* + 1)*a*. We can obtain some batch *B*_*k*_ whose processing time is greater than 2(*k* + 1)*a*, *k* ≠ *l*. Hence, *F*′ > *y*, which is a contradiction.

Therefore, a partition for the set *H* is obtained by letting the elements be corresponding to batch *B*_*l*_. Then it is easy to see that *q* = *h* + 1 and *h* batches *B*_1_,…, *B*_*h*_ form a solution to the 3-PP instance. The proof is concluded.

Theorem 4 indicates that the existence of a polynomial time algorithm to solve the scheduling model is unlikely. Since the general problem is strongly NP-hard, we next consider two special cases of the problem.

## 4. The Problem *Vm* → batch|*p*_*j*_ = *p*, *w*_*j*_|∑*C*_*j*_ + *X*(*x*)

We now turn our attention to a special case with identical processing time condition on the machine. In this section, we prove that the problem *Vm* → batch|*p*_*j*_ = *p*, *w*_*j*_|∑*C*_*j*_ + *X*(*x*) is ordinarily NP-hard by a reduction of the Partition Problem [13] which is a known NP-hard problem and provides a dynamic programming algorithm in pseudopolynomial time.

Suppose a feasible schedule *π* = {*B*_1_, *B*_2_,…*B*_*x*_} with *x* ∈ {⌈*n*/*c*⌉,…, *n*} batches for the problem *Vm* → batch|*p*_*j*_ = *p*, *w*_*j*_|∑*C*_*j*_ + *X*(*x*). We now present some properties for the problem.

**Lemma 5**

For* Vm*→batch|*p*_*j*_ = *p*, *w*_*j*_|∑*C*_*j*_ + *X*(*x*), there exists an optimal schedule π*such that all orders assigned into the same vehicle are scheduled in the nondecreasing sequence of their transportation times.

**Proof**

Assume that orders *J*_*i*_ and *J*_*j*_ are assigned to the same vehicle. *J*_*i*_ is followed by *J*_*j*_ immediately such that *t*_*i*_ ≥ *t*_*j*_ in π*. Let *π*′ be a schedule obtained by swapping *J*_*i*_ and *J*_*j*_. In *π*′, it is easy to see that *r*_*i*_ > *r*_*j*_′ and *r*_*i*_′ = *r*_*j*_. Regardless of whether *J*_*i*_ and *J*_*j*_ are processed in the same batch or not, the starting times of *J*_*i*_ and *J*_*j*_ on the machine cannot increase. We have *F*(π*) ≥ *F*(*π*′).

**Lemma 6**

For any scheduleof the problem* Vm*→batch|*p*_*j*_ = *p*, *w*_*j*_|∑*C*_*j*_ + *X*(*x*), the starting time of batch *B*_*l*_ satisfies *S*_*l*+1_ ≥ *S*_*l*_ + *p* for *l* = 1,2,…, *x*.

**Proof**

It is easily obtained based on Lemma 3.

**Lemma 7**

For any schedule of the problem* Vm*→batch|*p*_*j*_ = *p*, *w*_*j*_ | ∑*C*_*j*_ + *X*(*x*), *B*_*l*_ contains all orders which have arrived at the machine if the number of the orders is no more than the capacity *c* in the time interval (*S*_*l*−1_, *S*_*l*_].

**Proof**

Assume that the batches are numbered in accordance with their start times, and the number of orders in each batch is smaller than the machine capacity *c*. Suppose that *J*_*j*_ is the first order assigned in *B*_*l*+1_ in π*, and the arrival time of *J*_*j*_ on the machine satisfies *r*_*j*_ ≤ *S*_*l*_. Let *π*′ be a new schedule obtained by simply assigning *J*_*j*_ to *B*_*l*_. Then *C*_*j*_′ = *S*_*l*_ + *p* < *S*_*l*+1_ + *p* ≤ *C*_*j*_. The schedule of the remaining orders in *π*′ are the same as in π*. It is obvious that *F*(*π*′) ≤ *F*(π*). We can see that there exists an optimal schedule in which all batches consist of a number of orders which finish processing contiguously.

We will show that the problem with equal processing times is NP-hard by a reduction from the Partition Problem.

**Theorem 8**

The problem* Vm*→batch|*p*_*j*_ = *p*, *w*_*j*_|∑*C*_*j*_ + *X*(*x*) is NP-hard even if *m* = 2.

**Proof**

We prove this result by reducing the Partition Problem to* Vm*→batch|*p*_*j*_ = *p*, *w*_*j*_|∑*C*_*j*_ + *X*(*x*). The Partition Problem (PP) can be stated as follows: given *h* items, *H* = {1,2,…, *h*}, each item *i* ∈ *H* has a positive integer size *a*_*i*_, such that ∑_*i*=1_^*h*^ *a*_*i*_ = 2*a*, for some integer *a*. The question asked is whether there are two disjoint subsets *H*_1_ and *H*_2_, such that ∑_*i*∈*H*_1__ *a*_*i*_ = ∑_*i*∈*H*_2__ *a*_*i*_ = *a*.

We construct a corresponding instance of the problem* Vm*→batch|*p*_*j*_ = *p*, *w*_*j*_|∑*C*_*j*_ + *X*(*x*) as follows.

*Number of Orders*. Consider

(8) $$\begin{array}{c} n=2h. \end{array}$$

*Transportation Times*. Consider

(9) $$\begin{array}{c} t_{j}=a_{j},\;j\in H=\left\{1,2,\ldots ,h\right\};\\ t_{j}=0,\;j\in Y=\left\{h+1,h+2,\ldots ,2h\right\}. \end{array}$$

* Processing Times*. Consider

(10) $$\begin{array}{c} p_{j}=a,\;\;j=1,2,\ldots ,2\;h. \end{array}$$

* Limited Waiting Time*. Conider

(11) $$\begin{array}{c} W=a. \end{array}$$

*Processing Cost Function*. Consider

(12) $$\begin{array}{c} X\left(x\right)=3ahx. \end{array}$$

*Machine Capacity*. Consider

(13) $$\begin{array}{c} c=h. \end{array}$$

* Threshold Value*. Consider

(14) $$\begin{array}{c} y=9ah. \end{array}$$

First, it is easy to see that, in a solution to this instance of* Vm*→batch|*p*_*j*_ = *p*, *w*_*j*_|∑*C*_*j*_ + *X*(*x*) with the objective value not exceeding *y*. We first prove the following property: since *t*_*h*+1_ = ⋯ = *t*_2*h*_ = 0, all the orders of *Y* as the first batch must be processed on the machine in the time interval [0,* a*]. Now, we prove that there is a solution to the constructed instance of* Vm*→batch|*p*_*j*_ = *p*, *w*_*j*_|∑*C*_*j*_ + *X*(*x*) with total cost not exceeding *y* if and only if there is a solution to the PP instance.

→If there is a solution to the PP instance, we show that there is a schedule for the above-constructed instance with an objective value of no more than *y*. Let *H*_1_ and *H*_2_ be subsets of *H* that solves the PP instance. Vehicle 1 and Vehicle 2 transport the orders of *H*_1_ and the orders of *H*_2_, respectively. Let *T*_*u*_ be the total running time of vehicle* u*, for *u* = 1,2. Since ∑_*j*∈*H*_1__ *t*_*j*_ = ∑_*j*∈*H*_2__ *t*_*j*_ = *a*, we have *T*_1_ = *T*_2_ = *a*. In fact, the completion time of the first batch on the machine is *a*. We can obtain that the waiting time of each order on the machine is not greater than *a*. Let *B*_1_ = *Y* and *B*_2_ = *H*. It is easy to see that *b*_1_ = *b*_2_ = *h*, and the total flow time of all the orders is 3*ha*. Hence, the objective is* y*.

←Given a schedule with an objective value not exceeding* y*, then there must be a solution to the PP instance. We can obtain that all the orders in *H* are processed in the second batch on the machine; that is, all the orders are divided into two batches and *b*_1_ = *b*_2_ = *h*. Suppose that the number of the batches is greater than 2. Note that even if the waiting time constraint is ignored, the objective value is *F* ≥ *ah* + ∑_*j*∈*H*_ *C*_*j*_ + 9*ah* > *y*, which is a contradiction. Thus, *π* = {*B*_1_, *B*_2_} and *B*_1_ = *Y* and *B*_2_ = *H*.

If *T*_1_ = ∑_*j*∈*H*_1__ *t*_*j*_ < *a*, then it implies *T*_2_ = ∑_*j*∈*H*_2__ *t*_*j*_ > *a*. Hence, the starting time of batch *B*_2_ is *S*_2_ = *T*_2_. It implies that the total flow time is ∑*C*_*j*_ = *ah* + (*S*_2_ + *a*)*h* > 3*ah*. We have *F* > *y*, which is a contradiction. Thus, ∑_*j*∈*H*_1__ *a*_*j*_ = ∑_*j*∈*H*_2__ *a*_*j*_ = *a*.

Let *T* = ∑_*j*=1_^*n*^ *t*_*j*_. In the following, we derive a dynamic programming algorithm in pseudopolynomial time to solve the problem* Vm*→batch|*p*_*j*_ = *p*, *w*_*j*_|∑*C*_*j*_ + *X*(*x*).

**Algorithm 1 D1**

Index the orders in the nondecreasing transportation time sequence; that is, *t*_1_ ≤ *t*_2_ ≤ ⋯≤*t*_*n*_.

Define *H*_*R*_(*j*, *i*, *l*, *T*_1_, *T*_2_,…, *T*_*m*_) and *f*_*R*_(*j*, *i*, *l*, *T*_1_, *T*_2_,…, *T*_*m*_) as the minimum objective value and the minimum total flow time of a partial schedule of orders *J*_1_,…, *J*_*j*_, respectively, where orders *J*_1_,…, *J*_*j*_ have finished transporting and processing by using *l* batches on the machine, and the current batch *B*_*l*_ contains orders *J*_*i*_,…, *J*_*j*_.

*Initial Conditions*. Consider

(15) Hx(j,i,l,T1,T2,…,Tm) ={0,j=0,i=0,l=0,T1=T2=⋯Tm=0∞,otherwise,

 for *S*_1_ ≥ *t*_1_, *l* = 1,2,…, *x*, *x* ∈ {⌈*n*/*c*⌉,…, *n*}, and *T*_*u*_ = 0,1,…, *T*.

*Recursive Relations*. Consider

(16) fx(j,i,l,T1,T2,…,Tm)={min⁡1≤u≤m{fx(j−1,i,l,T1,…,Tu−tj,…,Tm)+Cj}if  bl<c∞  if  bl≥c,

where *C*_*j*_ = {*S*_*l*_ + *p* | *S*_*l*−1_ < *T*_*u*_ ≤ *S*_*l*_ ≤ *T*_*u*_′ + *W*, *l* = 1,2,…, *x*}, for *S*_*l*_ + *p* ≤ *S*_*l*+1_ ≤ *T*_*u*_′ + *W*,  *l* = 1, 2, …, *x*, *x* ∈ {⌈*n*/*c*⌉,…, *n*}, and *T*_*u*_′ = min⁡_*S*_*l*−1_<*T*_*u*_≤*S*_*l*__{*T*_*u*_}.

*Optimal Solution*. Consider

(17) $$\begin{array}{c} F^{*}=\min\left\{H_{x}\left(n,n+1,l,T_{1},T_{2},\ldots ,T_{m}\right)+X\left(x\right)\right\}. \end{array}$$

**Theorem 9**

Algorithm D1 can find an optimal schedule for the problem* Vm*→batch|*p*_*j*_ = *p*, *w*_*j*_|∑*C*_*j*_ + *X*(*x*) in* O*(*mn* ^3^ *T* ^*m*−1^ *W*) time.

**Proof**

Based on Lemma 5, there exists an optimal schedule with orders assigned to each vehicle in the nondecreasing transportation time sequence. If order *J*_*j*_ is assigned to vehicle* u*, then its starting time on the machine satisfies *r*_*j*_ + *W* ≤ *T*_*u*_. If the number of orders in the current batch *B*_*l*_ is less than* c*, order *J*_*j*_ can be assigned into *B*_*l*_. The corresponding flow time increases *S*_*l*_ + *p*. Based on Lemma 3, we have *S*_*l*−1_ < *T*_*u*_ ≤ *S*_*l*_ ≤ *T*_*u*_′ + *W*. This shows that Algorithm D1 can find optimal solution for the problem.

The time complexity of the algorithm can be established as follows. We observe that *i*, *j*, *l* ≤ *n*, *T*_*u*_ ≤ *T*, and *m* − 1 of the value *T*_1_, *T*_2_,…, *T*_*m*_ are independent. Thus, the number of different states of the recursive relations is at most* n* ^3^ *T* ^*m*−1^. For each state, each use requires *O*(*mW*). Therefore, the overall time complexity of Algorithm D1 is* O*(*mn* ^3^ *T* ^*m*−1^ *W*).

The existence of such a pseudopolynomial time algorithm for a NP-hard problem means that the problem is NP-hard in the ordinary sense. We have the following theorem.

**Theorem 10**

The problem* Vm*→batch|*p*_*j*_ = *p*, *w*_*j*_|∑*C*_*j*_ + *X*(*x*) is ordinarily NP-hard.

## 5. The Problem *Vm* → batch | *p*_*j*_ = *p*, *t*_*j*_ = *t*, *w*_*j*_ | ∑*C*_*j*_ + *X*(*x*)

In this section, we consider a special case with identical processing times and identical transportation times and derive a polynomial-time algorithm to solve it. It is evident that the problem reduces to an optimal batching problem on the machine for the special case. Note that *x* is a decision variable denoting the number of batches on the machine. Note that *n*_0_ = ⌈*n*/*m*⌉ and *g* = *n*_0_ *m* − *n*; let *n*_*u*_ be the number of orders transported on vehicle *u* under a specific schedule. Note that *n*_0_′ = ⌈*n*/*c*⌉ and *n*_0_′ ≤ *x* ≤ *n*_0_, if *c* ≥ *m*. Hence, the number of batches *x* on the machine can be numbered by *n*_0_′, *n*_0_′ + 1,…, *n*_0_. Otherwise, *n*_0_ ≤ *x* ≤ *n*_0_′ if *c* < *m*. Without loss of generality, assume that *c* ≥ *m* in the following discussion. Let π* = (*B*_1_, *B*_2_,…*B*_*L*_) be an optimal schedule with given *x* = *L* batches for the problem* Vm*→batch|*p*_*j*_ = *p*, *t*_*j*_ = *t*, *w*_*j*_ | ∑*C*_*j*_ + *X*(*x*). Then we have the following lemma.

**Lemma 11**

For the problem* Vm*→batch|*p*_*j*_ = *p*, *t*_*j*_ = *t*, *w*_*j*_|∑*C*_*j*_ + *X*(*x*), there exists an schedule with *L* batches in which (1) *n*_0_ − 1 ≤ *n*_*u*_ ≤ *n*_0_, *u* = 1,2,…, *m*, (2) ${\overset{-}{b}}_{l}=b_{l}/m$, ${\overset{-}{b}}_{l}$ is integral, and *l* = 1,2,…, *L* − 1, and (3) *S*_*l*+1_ = max⁡{*r*_*j*_, *S*_*l*_ + *p*}, where *r*_*j*_ denotes the arrival time of some *m* jobs on the machine, *l* = 1,2,…, *L* − 1.

**Proof**

(1) Based on Lemma 2, each vehicle has no inserted idle time during transporting. Assume that there exists an optimal schedule π* in which the condition is not satisfied. Then there must be a pair of vehicles *u* and *v* such that *n*_*u*_ ≥ *n*_*v*_ + 2 and the last order on vehicle *u* is processed in the last batch. If the last order on vehicle *u* is moved to the last position on vehicle* v*, then the objective value will not increase. By repeating this process, we can obtain a desired optimal schedule.

(2) According to Lemma 7, in the optimal schedule, *B*_*l*_ contains all orders which finish transportation in the time interval (*S*_*l*−1_, *S*_*l*_], if the number of orders in a batch is no more than *c*. Since *t*_*j*_ = *t* for each order *J*_*j*_,* m* orders arrive at the machine together. A batch contains *m* orders or* km *(*km* ≤ *c*) orders, if their waiting times are not greater than the limited value *W*. Hence, ${\overset{-}{b}}_{l}$ is integral, for *l* = 1,2,…, *L* − 1.

(3) It is trivial according to Lemma 3.

Based on these results, we can easily construct an optimal schedule for the problem.

**Algorithm 2 D2**

Calculate *n*_0_ = ⌈*n*/*m*⌉, *n*_0_′ = ⌈*n*/*c*⌉.

For each *L* = *n*_0_′, *n*_0_′ + 1,…, *n*_0_, compute ${\overset{-}{b}}_{l}={\overset{-}{b}}_{0}-1$, *l* = 1, ⋯, *h*, ${\overset{-}{b}}_{l}={\overset{-}{b}}_{0}$, and *l* = *h* + 1,…, *L*, where ${\overset{-}{b}}_{0}=\left\lceil n_{0}/L\right\rceil$ and $h={\overset{-}{b}}_{0}L-n_{0}$.

Consider the following two cases.

**Case 1**

Consider *h* = 0.

If $({\overset{-}{b}}_{0}-1)t>W$ or $m{\overset{-}{b}}_{0}>c$, then the constructed schedule is not feasible, and denote *F*(*π*_L_*^  ^) = *∞*. If $({\overset{-}{b}}_{0}-1)t\leq W$ and $m{\overset{-}{b}}_{0}\leq c$, then $S_{1}={\overset{-}{b}}_{0}t$. According to Lemma 6, there must be ${\overset{-}{b}}_{0}tl+t+W\geq S_{l}+p$. Otherwise, it is not feasible. Based on Lemma 7, we can obtain

(18) $$\begin{array}{c} S_{l+1}=\max\left\{{\overset{-}{b}}_{0}t\left(l+1\right),S_{l}+p\right\},\;l=1,2,\ldots ,L-1. \end{array}$$

The associated objective value can be derived as

(19) F1(πL∗)=min⁡{∑l=1Lmb−0(Sl+p)}−g(SL+p)+X(L).

**Case 2**

Consider  *h* > 0.

If $({\overset{-}{b}}_{0}-2)t>W$ or $m{\overset{-}{b}}_{0}>c$, then it is not feasible, and denote *F*(*π*_L_*^  ^) = *∞*. If $({\overset{-}{b}}_{0}-2)t\leq W$ and $m{\overset{-}{b}}_{0}\leq c$, then we can show $S_{1}=({\overset{-}{b}}_{0}-1)t$. According to Lemma 6, we have

(20) $$\begin{array}{c} \left({\overset{-}{b}}_{0}-1\right)tl+t+W\geq S_{l}+p\;\text{if}\;\;l\leq h\\ \left({\overset{-}{b}}_{0}-1\right)th+{\overset{-}{b}}_{0}t\left(l-h\right)+t+W\geq S_{l}+p\;\text{if}\;\;l>h. \end{array}$$

Otherwise, the constructed schedule is not feasible, and denote *F*(*π*_L_*^  ^) = *∞*.

According to Lemma 6, we have

(21) Sl+1=max⁡⁡{(b−0−1)t(l+1),Sl+p} if  l≤h−1Sl+1=max⁡⁡{(b−0−1)th+b−0t(l+1−h),Sl+p} if  l≥h,                       l=1,2,…,L−1.

The associated objective value can be derived as

(22) F2(πL∗  ) =min⁡{∑l=1hm(b−0−1)(Sl+p)+∑l=h+1Lmb−0(Sl+p)}  −g(SL+p)+X(L).

Thus, the objective value is

(23) $$\begin{array}{c} F\left({\pi_{L}^{*}}^{\;}\right)=\min\left\{F_{1}\left({\pi_{L}^{*}}^{\;}\right),F_{2}\left({\pi_{L}^{*}}^{\;}\right)\right\}. \end{array}$$

**Theorem 12**

The problem* Vm*→batch|*p*_*j*_ = *p*,   *t*_*j*_ = *t*, *w*_*j*_|∑*C*_*j*_ + *X*(*x*) can be solved by Algorithm D2 in *O*(*n*/*m*) time.

**Proof**

Equations (18) and (21) present that the machine can start to process *B*_*l*+1_ as soon as the orders assigned into *B*_*l*+1_ have arrived at the machine and the machine has finished processing *B*_*l*_. The local optimal solution in two cases can be obtained by (19) and (22). The optimal solution is derived as (23). Hence, the optimal schedule to this special case can be obtained by repeating the above procedure for *n*_0_′ ≤ *L* ≤ *n*_0_ and selecting the best candidate among the solutions generated. This shows that Algorithm D2 can find optimal solution. It is clear that the overall time complexity of Algorithm D2 is *O*(*n*/*m*).

We now demonstrate the above algorithm with the following numerical example.

**Example 13**

Consider the instance with 11 orders, *m* = 2,  *t* = 1,  *c* = 4,  *W* = 2,  *p* = 2, and *X*(*x*) = 5*x*. Based on the above method, we know *n*_0_ = 6, *g* = 1, and *n*_0_′ = 2. Then, *L* = 2, 3, 4, 5, 6. We have the following results:

 when *L* = 2, we have ${\overset{-}{b}}_{0}=3$, *h* = 0,  *S*_1_ = 3, and *F*(π*) = 80;
 when *L* = 3, we have ${\overset{-}{b}}_{0}=2$, *h* = 0,  *S*_1_ = 2, and *F*(π*) = 79;
 when *L* = 4, we have ${\overset{-}{b}}_{0}=2$, *h* = 2,  *S*_1_ = 1, and *F*(*π*_L_*) = 101;
 when *L* = 5, we have ${\overset{-}{b}}_{0}=2,h=4$,$\;\;{\overset{-}{b}}_{0}t\cdot 3+t+W<S_{3}+p$, and *F*(*π*_L_*) = *∞*;
 when *L* = 6, we have ${\overset{-}{b}}_{0}=1,h=0$,$\;\;{\overset{-}{b}}_{0}t\cdot 3+t+W<S_{3}+p$, and *F*(*π*_L_*) = *∞*.

Therefore, we can obtain an optimal schedule π* for *L* = 3 with *B*_1_ = {*J*_1_, *J*_2_, *J*_3_, *J*_4_}, *B*_2_ = {*J*_5_, *J*_6_, *J*_7_, *J*_8_}, and *B*_3_ = {*J*_9_, *J*_10_, *J*_11_}.

## 6. Concluding Remarks 

In this paper, we address a coordinated scheduling problem with order transportation before processing and parallel-batch production under the limited waiting time consideration. Our goal is to optimize the sum of the total flow time and the total processing cost. First, we prove that the general problem is NP-hard in the strong sense. We also demonstrate that the problem with equal processing times on the machine is NP-hard. Furthermore, a dynamic programming algorithm in pseudopolynomial time is provided to prove its ordinarily NP-hardness. The problem with equal processing times and equal transportation times for each order can be solved in polynomial time through taking into account the properties of the special case. Our work has practical implications for the coordination of batch-production and transportation to improve the overall system performance in logistics and operations management. Simultaneously, another important implication of our paper may provide a vital approach to deal with the waiting time constraint applied to reduce the raw material consumption and improve the machine utilization.

There are several possible extensions to this research. First, it is interesting to investigate the problems with other objective functions such as minimizing the makespan or minimizing maximum order tardiness/earliness. Another interesting issue is to develop effective heuristics to solve the general problem and investigate polynomial time algorithms for other special cases.

## Figures and Tables

**Table 1:** 

Order	1	2	3	4	5	6	7	8
*t*_*j*_	1	1	2	2	2	3	4	4
*p*_*j*_	2	3	4	3	3	3	4	4
